# *catena*-Poly[[(μ-3-hy­droxy­benzoato-κ^3^ *O* ^1^,*O* ^1′^:*O* ^3^)(μ-3-hy­droxy­benzoato-κ^3^ *O* ^1^,*O* ^1′^:*O* ^1^)(isonicotinamide-κ*N* ^1^)­lead(II)] monohydrate]

**DOI:** 10.1107/S1600536812004357

**Published:** 2012-02-10

**Authors:** Ibrahim Göker Zaman, Nagihan Çaylak Delibaş, Hacali Necefoğlu, Tuncer Hökelek

**Affiliations:** a Department of Chemistry, Kafkas University, 36100 Kars, Turkey; b Department of Physics, Sakarya University, 54187 Esentepe, Sakarya, Turkey; c Department of Physics, Hacettepe University, 06800 Beytepe, Ankara, Turkey

## Abstract

In the crystal of the title polymeric compound, {[Pb(C_7_H_5_O_3_)_2_(C_6_H_6_N_2_O)]·H_2_O}_*n*_, the Pb^II^ ion is chelated by two carboxyl­ate groups of 3-hy­droxy­benzoate (HB) anions, and coordinated by one isonicotinamide mol­ecule; a carboxyl­ate O atom and a hy­droxy O atom from adjacent HB anions bridge the Pb^II^ ion to form polymeric chains along [100], in which the Pb^II^ ion is in an irregular seven-coordination geometry. The carboxyl­ate groups of the HB ions are slightly twisted away from the attached benzene rings by 2.84 (15) and 4.8 (2)°. The planes of the two benzene rings of the HB ions are oriented with respect to each other at a dihedral angle of 84.41 (8)°. In the crystal, adjacent polymeric chains inter­act *via* O—H⋯O, N—H⋯O and weak C—H⋯O hydrogen bonds. The solvent water mol­ecule links with the polymeric chains *via* O—H⋯O hydrogen bonding. π–π stacking between the benzene and pyridine rings and between the benzene rings [centroid–centroid distances = 3.731 (2) and 3.353 (2) Å] are present in the crystal.

## Related literature

For niacin, see: Krishnamachari (1974 ▶). For *N*,*N*-diethyl­nicotinamide, see: Bigoli *et al.* (1972 ▶). For related structures, see: Greenaway *et al.* (1984 ▶); Hökelek & Necefoğlu (1996 ▶); Hökelek, Yılmaz *et al.* (2009 ▶); Hökelek, Dal *et al.* (2009*a* ▶,*b* ▶,*c* ▶, 2010 ▶); Hökelek, Süzen *et al.* (2010 ▶); Hökelek *et al.* (2011 ▶).
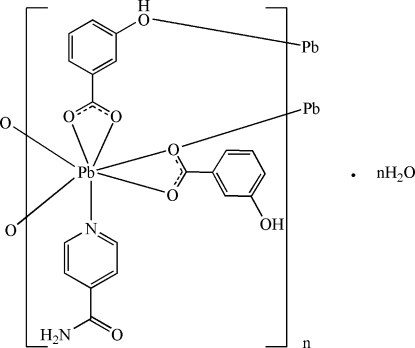


## Experimental

### 

#### Crystal data

[Pb(C_7_H_5_O_3_)_2_(C_6_H_6_N_2_O)]·H_2_O
*M* *_r_* = 621.56
Triclinic, 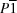

*a* = 7.3626 (2) Å
*b* = 12.1382 (3) Å
*c* = 12.1789 (3) Å
α = 67.165 (2)°
β = 74.192 (3)°
γ = 88.921 (3)°
*V* = 960.53 (5) Å^3^
*Z* = 2
Mo *K*α radiation
μ = 8.84 mm^−1^
*T* = 100 K
0.26 × 0.14 × 0.13 mm

#### Data collection

Bruker Kappa APEXII CCD area-detector diffractometer
Absorption correction: multi-scan (*SADABS*; Bruker, 2005 ▶) *T*_min_ = 0.395, *T*_max_ = 0.603
17040 measured reflections
4783 independent reflections
4652 reflections with *I* > 2σ(*I*)
*R*_int_ = 0.032

#### Refinement

*R*[*F* ^2^ > 2σ(*F* ^2^)] = 0.017
*wR*(*F* ^2^) = 0.050
*S* = 1.19
4783 reflections
304 parameters
4 restraints
H atoms treated by a mixture of independent and constrained refinement
Δρ_max_ = 0.94 e Å^−3^
Δρ_min_ = −1.27 e Å^−3^

### 

Data collection: *APEX2* (Bruker, 2007 ▶); cell refinement: *SAINT* (Bruker, 2007 ▶); data reduction: *SAINT*; program(s) used to solve structure: *SHELXS97* (Sheldrick, 2008 ▶); program(s) used to refine structure: *SHELXL97* (Sheldrick, 2008 ▶); molecular graphics: *ORTEP-3 for Windows* (Farrugia, 1997 ▶); software used to prepare material for publication: *WinGX* (Farrugia, 1999 ▶) and *PLATON* (Spek, 2009 ▶).

## Supplementary Material

Crystal structure: contains datablock(s) I, global. DOI: 10.1107/S1600536812004357/xu5463sup1.cif

Structure factors: contains datablock(s) I. DOI: 10.1107/S1600536812004357/xu5463Isup2.hkl

Additional supplementary materials:  crystallographic information; 3D view; checkCIF report

## Figures and Tables

**Table 1:** Selected bond lengths (Å)

Pb1—N1	2.564 (2)
Pb1—O1	2.753 (2)
Pb1—O2	2.317 (2)
Pb1—O3^i^	2.899 (2)
Pb1—O4	2.742 (2)
Pb1—O5	2.344 (2)
Pb1—O5^ii^	2.954 (2)

Symmetry codes: (i) 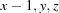; (ii) 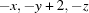.

**Table 2:** Hydrogen-bond geometry (Å, °)

*D*—H⋯*A*	*D*—H	H⋯*A*	*D*⋯*A*	*D*—H⋯*A*
N2—H21⋯O4^iii^	0.84 (4)	2.21 (4)	3.046 (3)	175 (4)
N2—H22⋯O8^iv^	0.85 (4)	2.06 (4)	2.880 (5)	162 (4)
O3—H31⋯O7^v^	0.86 (5)	1.81 (5)	2.646 (3)	166 (4)
O6—H61⋯O1^vi^	0.73 (6)	2.05 (6)	2.755 (4)	161 (6)
O8—H81⋯O6^vii^	0.89 (4)	2.02 (4)	2.846 (3)	154 (4)
O8—H82⋯O2^viii^	0.87 (5)	2.31 (5)	3.018 (3)	139 (5)
O8—H82⋯O3^ix^	0.87 (5)	2.44 (6)	3.054 (3)	128 (4)
C12—H12⋯O1^vi^	0.93	2.57	3.269 (4)	132
C15—H15⋯O5	0.93	2.46	3.060 (4)	122
C16—H16⋯O8^iv^	0.93	2.51	3.413 (4)	165

Symmetry codes: (iii) 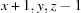; (iv) 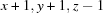; (v) 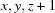; (vi) 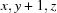; (vii) 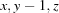; (viii) 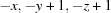; (ix) 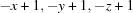.

**Fig. 1.**
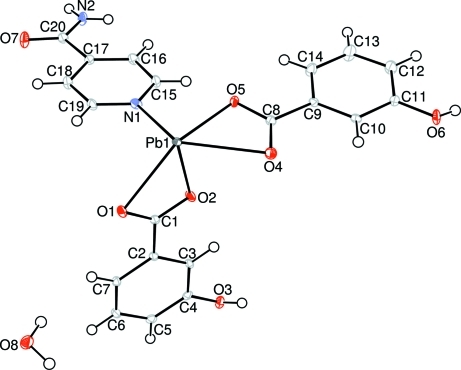
The asymmetric unit of the title compound with the atom-numbering scheme. Displacement ellipsoids are drawn at the 50% probability level.

**Fig. 2.**
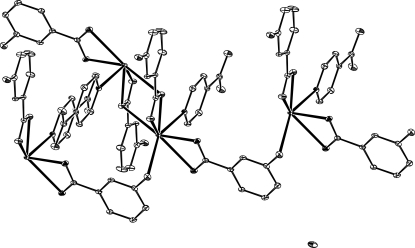
The polymeric structure.

## supplementary crystallographic information

### Comment

As a part of our ongoing investigation on transition metal complexes of nicotinamide (NA), one form of niacin (Krishnamachari, 1974), and/or the nicotinic acid derivative *N*,*N*-diethylnicotinamide (DENA), an important respiratory stimulant (Bigoli *et al.*, 1972), the title compound was synthesized and its crystal structure is reported herein.

In the crystal structure of the polymeric title compound, (I), the Pb^II^ ion is chelated by two carboxyl groups of 3-hydroxybenzoate (HB) anions, and coordinated by one isonicotinamide (INA) molecule (Fig. 1); a carboxyl-O atom and a hydroxyl-O atom from adjacent HB anions bridge the Pb^II^ ion to form the polymeric complex, in which the Pb^II^ ion is in an irregular seven-coordination geometry (Fig. 2). The two HB ions act as bidentate ligands, while the INA is monodentate ligand (Fig. 1).

The average Pb-O bond length (Table 1) is 2.539 (2) Å, and the Pb atom is displaced out of the least-squares planes of the carboxylate groups (O1/C1/O2) and (O4/C8/O5) by -0.0641 (1) and -0.0110 (1) Å, respectively. In (I), the O1-Pb1-O2 and O4-Pb1-O5 angles are 51.00 (6) and 50.94 (6) °, respectively. The corresponding O-M-O (where M is a metal) angles are 51.10 (15)° and 51.95 (16)° in {[Pb(PEB)_2_(NA)].H_2_O}_n_ (Hökelek *et al.*, 2011), 51.09 (6)° and 51.71 (5)° in [Pb(PMB)_2_(NA)]_n_ (Hökelek, Dal *et al.*, 2010), 55.96 (4)° and 53.78 (4)° in [Cd_2_(DMAB)_4_(NA)_2_(H_2_O)_2_] (Hökelek, Süzen *et al.*, 2010), 52.91 (4)° and 53.96 (4)° in [Cd(FB)_2_(INA)_2_(H_2_O)].H_2_O (Hökelek, Yılmaz *et al.*, 2009), 60.70 (4)° in [Co(DMAB)_2_(INA)(H_2_O)_2_] (Hökelek, Dal *et al.*, 2009*a*), 58.45 (9)° in [Mn(DMAB)_2_(INA)(H_2_O)_2_] (Hökelek, Dal *et al.*, 2009*b*), 60.03 (6)° in [Zn(MAB)_2_(INA)_2_].H_2_O (Hökelek, Dal *et al.*, 2009*c*), 58.3 (3)° in [Zn_2_(DENA)_2_(HB)_4_].2H_2_O (Hökelek & 'Necefoğlu, 1996) [where NA, INA, DENA, HB, FB, MAB, PMB, PEB and DMAB are nicotinamide, isonicotinamide, *N*,*N*-diethylnicotinamide, 4-hydroxybenzoate, 4-formylbenzoate, 4-methylaminobenzoate, 4-methylbenzoate, 4-ethylbenzoate and 4-dimethylaminobenzoate, respectively] and 55.2 (1)° in [Cu(Asp)_2_(py)_2_] (where Asp is acetylsalicylate and py is pyridine) (Greenaway *et al.*, 1984).

The dihedral angles between the planar carboxylate groups and the adjacent benzene rings A (C2-C7) and B (C9-C14) are 2.84 (15) and 4.8 (2) °, respectively, while those between rings A, B, C (N1/C15-C19), D (Pb1/O1/O2/C1) and E (Pb1/O4/O5/C8) are A/B = 84.41 (8), A/C = 87.69 (7), B/C = 10.23 (9) and D/E = 80.12 (9)°.

In the crystal, adjacent polymeric chains interact *via* O—H···O, N—H···O and weak C—H···O hydrogen bonds; and the lattice water molecule links with the polymeric chains *via* O—H···O hydrogen bonding (Table 2), in which they may be effective in the stabilization of the structure. π···π Contacts between the benzene and pyridine rings and between the benzene rings, Cg3—Cg2^i^ and Cg1—Cg1^ii^ [symmetry codes: (i) 1 - x, 2 - y, -z, (ii) 1 - x, 1 - y, 1 - z, where Cg1, Cg2 and Cg3 are the centroids of the rings A (C2-C7), B (C9-C14) and C (N1/C15-C19), respectively] may further stabilize the structure, with centroid-centroid distances of 3.731 (2) and 3.353 (2) Å, respectively.

### Experimental

The title compound was prepared by the reaction of Pb(NO_3_)_2_ (1.656 g, 5 mmol) in H_2_O (100 ml) and INA (1.220 g, 10 mmol) in H_2_O (50 ml) with sodium 3-hydroxybenzoate (1.601 g, 10 mmol) in H_2_O (100 ml). The mixture was filtered and set aside to crystallize at ambient temperature for two weeks, giving yellow single crystals.

### Refinement

Atoms H81 and H82 (for H_2_O), H21 and H22 (for NH_2_) and H31 and H61 (for OH) were located in a difference Fourier map and were refined isotropically. The C-bound H-atoms were positioned geometrically with C—H = 0.93 Å and constrained to ride on their parent atoms, with *U*_iso_(H) = 1.2*U*_eq_(C).

### Crystal data

**Table:**

[Pb(C_7_H_5_O_3_)_2_(C_6_H_6_N_2_O)]·H_2_O	*Z* = 2
*M**_r_* = 621.56	*F*(000) = 596
Triclinic, *P*1	*D*_x_ = 2.149 Mg m^−^^3^
Hall symbol: -P 1	Mo *K*α radiation, λ = 0.71073 Å
*a* = 7.3626 (2) Å	Cell parameters from 9959 reflections
*b* = 12.1382 (3) Å	θ = 2.9–28.5°
*c* = 12.1789 (3) Å	µ = 8.84 mm^−^^1^
α = 67.165 (2)°	*T* = 100 K
β = 74.192 (3)°	Block, yellow
γ = 88.921 (3)°	0.26 × 0.14 × 0.13 mm
*V* = 960.53 (5) Å^3^	

### Data collection

**Table:**

Bruker Kappa APEXII CCD area-detector diffractometer	4783 independent reflections
Radiation source: fine-focus sealed tube	4652 reflections with *I* > 2σ(*I*)
Graphite monochromator	*R*_int_ = 0.032
φ and ω scans	θ_max_ = 28.5°, θ_min_ = 1.9°
Absorption correction: multi-scan (*SADABS*; Bruker, 2005)	*h* = −9→9
*T*_min_ = 0.395, *T*_max_ = 0.603	*k* = −16→16
17040 measured reflections	*l* = −16→16

### Refinement

**Table:**

Refinement on *F*^2^	Primary atom site location: structure-invariant direct methods
Least-squares matrix: full	Secondary atom site location: difference Fourier map
*R*[*F*^2^ > 2σ(*F*^2^)] = 0.017	Hydrogen site location: inferred from neighbouring sites
*wR*(*F*^2^) = 0.050	H atoms treated by a mixture of independent and constrained refinement
*S* = 1.19	*w* = 1/[σ^2^(*F*_o_^2^) + (0.0228*P*)^2^ + 0.6812*P*] where *P* = (*F*_o_^2^ + 2*F*_c_^2^)/3
4783 reflections	(Δ/σ)_max_ = 0.001
304 parameters	Δρ_max_ = 0.94 e Å^−^^3^
4 restraints	Δρ_min_ = −1.27 e Å^−^^3^

### Special details

**Table:**

Geometry. All esds (except the esd in the dihedral angle between two l.s. planes) are estimated using the full covariance matrix. The cell esds are taken into account individually in the estimation of esds in distances, angles and torsion angles; correlations between esds in cell parameters are only used when they are defined by crystal symmetry. An approximate (isotropic) treatment of cell esds is used for estimating esds involving l.s. planes.
Refinement. Refinement of F^2^ against ALL reflections. The weighted R-factor wR and goodness of fit S are based on F^2^, conventional R-factors R are based on F, with F set to zero for negative F^2^. The threshold expression of F^2^ > 2sigma(F^2^) is used only for calculating R-factors(gt) etc. and is not relevant to the choice of reflections for refinement. R-factors based on F^2^ are statistically about twice as large as those based on F, and R- factors based on ALL data will be even larger.

### Fractional atomic coordinates and isotropic or equivalent isotropic displacement parameters (Å^2^)

**Table:**

	*x*	*y*	*z*	*U*_iso_*/*U*_eq_	
Pb1	0.030913 (12)	0.835470 (8)	0.138812 (8)	0.00786 (4)	
O1	0.1928 (3)	0.62307 (19)	0.21830 (19)	0.0120 (4)	
O2	0.2876 (3)	0.79500 (18)	0.21917 (19)	0.0119 (4)	
O3	0.8367 (3)	0.7102 (2)	0.40132 (19)	0.0124 (4)	
H31	0.765 (6)	0.748 (4)	0.440 (4)	0.024 (10)*	
O4	0.0065 (3)	0.98903 (19)	0.25722 (19)	0.0136 (4)	
O5	0.1523 (3)	1.03718 (18)	0.05741 (18)	0.0110 (4)	
O6	0.0703 (3)	1.3836 (2)	0.3029 (2)	0.0136 (4)	
H61	0.111 (7)	1.442 (5)	0.294 (5)	0.042 (15)*	
O7	0.6149 (3)	0.7967 (2)	−0.4465 (2)	0.0172 (5)	
O8	−0.1509 (3)	0.1982 (2)	0.5265 (2)	0.0168 (4)	
H81	−0.066 (6)	0.260 (3)	0.473 (4)	0.057 (16)*	
H82	−0.127 (8)	0.204 (5)	0.590 (4)	0.062 (17)*	
N1	0.2801 (3)	0.8592 (2)	−0.0646 (2)	0.0106 (5)	
N2	0.7553 (4)	0.9791 (3)	−0.4941 (2)	0.0144 (5)	
H21	0.830 (5)	0.981 (4)	−0.561 (3)	0.039 (13)*	
H22	0.769 (6)	1.035 (3)	−0.471 (4)	0.028 (11)*	
C1	0.3015 (4)	0.6828 (3)	0.2424 (2)	0.0088 (5)	
C2	0.4561 (4)	0.6274 (2)	0.2976 (2)	0.0090 (5)	
C3	0.5721 (4)	0.6960 (3)	0.3251 (2)	0.0093 (5)	
H3	0.5529	0.7759	0.3098	0.011*	
C4	0.7168 (4)	0.6450 (3)	0.3754 (2)	0.0096 (5)	
C5	0.7482 (4)	0.5263 (3)	0.3954 (2)	0.0109 (5)	
H5	0.8481	0.4931	0.4262	0.013*	
C6	0.6310 (4)	0.4575 (3)	0.3697 (3)	0.0112 (5)	
H6	0.6502	0.3775	0.3853	0.013*	
C7	0.4846 (4)	0.5077 (3)	0.3203 (2)	0.0096 (5)	
H7	0.4063	0.4617	0.3026	0.012*	
C8	0.0990 (4)	1.0640 (3)	0.1532 (3)	0.0091 (5)	
C9	0.1525 (4)	1.1908 (3)	0.1322 (3)	0.0091 (5)	
C10	0.0883 (4)	1.2299 (3)	0.2284 (3)	0.0094 (5)	
H10	0.0149	1.1770	0.3063	0.011*	
C11	0.1341 (4)	1.3476 (3)	0.2076 (3)	0.0103 (5)	
C12	0.2455 (5)	1.4274 (3)	0.0911 (3)	0.0178 (6)	
H12	0.2756	1.5067	0.0771	0.021*	
C13	0.3106 (5)	1.3867 (3)	−0.0033 (3)	0.0222 (7)	
H13	0.3856	1.4392	−0.0808	0.027*	
C14	0.2653 (4)	1.2688 (3)	0.0164 (3)	0.0154 (6)	
H14	0.3101	1.2422	−0.0473	0.019*	
C15	0.4005 (4)	0.9580 (3)	−0.1397 (3)	0.0118 (5)	
H15	0.4060	1.0194	−0.1128	0.014*	
C16	0.5163 (4)	0.9719 (3)	−0.2555 (3)	0.0118 (5)	
H16	0.5959	1.0422	−0.3058	0.014*	
C17	0.5126 (4)	0.8796 (3)	−0.2960 (3)	0.0094 (5)	
C18	0.3894 (4)	0.7765 (3)	−0.2170 (3)	0.0127 (5)	
H18	0.3832	0.7127	−0.2406	0.015*	
C19	0.2766 (4)	0.7705 (3)	−0.1032 (3)	0.0116 (5)	
H19	0.1946	0.7016	−0.0512	0.014*	
C20	0.6331 (4)	0.8831 (3)	−0.4197 (3)	0.0110 (5)	

### Atomic displacement parameters (Å^2^)

**Table:**

	*U*^11^	*U*^22^	*U*^33^	*U*^12^	*U*^13^	*U*^23^
Pb1	0.00963 (6)	0.00600 (6)	0.00604 (6)	−0.00059 (4)	−0.00152 (4)	−0.00083 (4)
O1	0.0153 (9)	0.0070 (10)	0.0142 (10)	−0.0003 (7)	−0.0053 (8)	−0.0041 (8)
O2	0.0148 (9)	0.0063 (10)	0.0158 (10)	0.0004 (7)	−0.0071 (8)	−0.0038 (8)
O3	0.0131 (9)	0.0132 (11)	0.0127 (10)	−0.0004 (8)	−0.0023 (8)	−0.0078 (9)
O4	0.0191 (10)	0.0097 (11)	0.0091 (9)	−0.0018 (8)	−0.0011 (8)	−0.0024 (8)
O5	0.0159 (9)	0.0092 (10)	0.0087 (9)	0.0010 (7)	−0.0028 (7)	−0.0051 (8)
O6	0.0170 (10)	0.0095 (11)	0.0140 (10)	−0.0022 (8)	0.0014 (8)	−0.0082 (9)
O7	0.0193 (10)	0.0158 (12)	0.0166 (11)	−0.0039 (8)	0.0024 (8)	−0.0113 (9)
O8	0.0231 (11)	0.0151 (12)	0.0097 (10)	−0.0060 (9)	−0.0013 (8)	−0.0042 (9)
N1	0.0095 (10)	0.0100 (12)	0.0088 (11)	0.0013 (9)	0.0002 (8)	−0.0019 (9)
N2	0.0185 (12)	0.0136 (13)	0.0096 (12)	−0.0024 (10)	0.0021 (9)	−0.0071 (10)
C1	0.0103 (11)	0.0079 (13)	0.0056 (12)	−0.0003 (9)	0.0005 (9)	−0.0019 (10)
C2	0.0117 (12)	0.0067 (13)	0.0059 (12)	−0.0004 (9)	−0.0002 (9)	−0.0013 (10)
C3	0.0122 (12)	0.0058 (13)	0.0068 (12)	−0.0008 (9)	0.0012 (9)	−0.0017 (10)
C4	0.0101 (12)	0.0117 (14)	0.0047 (11)	−0.0016 (10)	0.0007 (9)	−0.0027 (10)
C5	0.0124 (12)	0.0118 (14)	0.0058 (12)	0.0014 (10)	−0.0003 (9)	−0.0023 (11)
C6	0.0157 (13)	0.0072 (13)	0.0091 (12)	0.0014 (10)	−0.0017 (10)	−0.0027 (11)
C7	0.0121 (12)	0.0075 (13)	0.0083 (12)	−0.0011 (10)	−0.0011 (9)	−0.0033 (10)
C8	0.0087 (11)	0.0084 (13)	0.0094 (12)	0.0001 (9)	−0.0023 (9)	−0.0029 (11)
C9	0.0109 (12)	0.0067 (13)	0.0088 (12)	−0.0001 (9)	−0.0031 (10)	−0.0020 (10)
C10	0.0111 (12)	0.0063 (13)	0.0072 (12)	−0.0012 (10)	−0.0011 (9)	0.0001 (10)
C11	0.0113 (12)	0.0105 (14)	0.0102 (13)	0.0017 (10)	−0.0033 (10)	−0.0051 (11)
C12	0.0259 (15)	0.0104 (15)	0.0129 (14)	−0.0044 (12)	−0.0012 (12)	−0.0030 (12)
C13	0.0323 (17)	0.0158 (17)	0.0103 (14)	−0.0101 (13)	0.0053 (12)	−0.0039 (13)
C14	0.0206 (14)	0.0138 (15)	0.0094 (13)	−0.0041 (11)	−0.0001 (11)	−0.0046 (12)
C15	0.0135 (12)	0.0093 (14)	0.0128 (13)	0.0011 (10)	−0.0022 (10)	−0.0058 (11)
C16	0.0112 (12)	0.0094 (14)	0.0113 (13)	−0.0018 (10)	−0.0002 (10)	−0.0021 (11)
C17	0.0092 (11)	0.0096 (14)	0.0087 (12)	0.0005 (10)	−0.0017 (10)	−0.0033 (11)
C18	0.0132 (12)	0.0109 (14)	0.0145 (14)	0.0007 (10)	−0.0020 (10)	−0.0069 (12)
C19	0.0136 (12)	0.0068 (13)	0.0119 (13)	−0.0019 (10)	−0.0019 (10)	−0.0022 (11)
C20	0.0113 (12)	0.0107 (14)	0.0097 (13)	0.0004 (10)	−0.0014 (10)	−0.0037 (11)

### Geometric parameters (Å, º)

**Table:**

Pb1—N1	2.564 (2)	C5—C4	1.390 (4)
Pb1—O1	2.753 (2)	C5—C6	1.386 (4)
Pb1—O2	2.317 (2)	C5—H5	0.9300
Pb1—O3^i^	2.899 (2)	C6—H6	0.9300
Pb1—O4	2.742 (2)	C7—C2	1.393 (4)
Pb1—O5	2.344 (2)	C7—C6	1.393 (4)
Pb1—O5^ii^	2.954 (2)	C7—H7	0.9300
O1—C1	1.253 (3)	C9—C14	1.390 (4)
O2—C1	1.287 (3)	C8—C9	1.501 (4)
O3—C4	1.370 (3)	C10—C9	1.394 (4)
O3—H31	0.86 (4)	C10—H10	0.9300
O4—C8	1.247 (3)	C11—C10	1.382 (4)
O5—C8	1.288 (3)	C11—C12	1.397 (4)
O6—C11	1.361 (3)	C12—C13	1.389 (4)
O6—H61	0.73 (5)	C12—H12	0.9300
O7—C20	1.234 (4)	C13—C14	1.387 (4)
O8—H81	0.89 (2)	C13—H13	0.9300
O8—H82	0.87 (2)	C14—H14	0.9300
N1—C15	1.344 (4)	C15—H15	0.9300
N1—C19	1.334 (4)	C16—C15	1.382 (4)
N2—C20	1.328 (4)	C16—H16	0.9300
N2—H21	0.844 (19)	C17—C16	1.391 (4)
N2—H22	0.843 (19)	C17—C18	1.396 (4)
C1—C2	1.495 (4)	C17—C20	1.510 (4)
C3—C2	1.390 (4)	C18—H18	0.9300
C3—C4	1.390 (4)	C19—C18	1.383 (4)
C3—H3	0.9300	C19—H19	0.9300
			
O2—Pb1—O1	51.00 (6)	C2—C7—H7	120.2
O2—Pb1—O4	77.71 (7)	C6—C7—H7	120.2
O2—Pb1—O5	85.33 (7)	O4—C8—O5	121.9 (3)
O2—Pb1—N1	83.32 (7)	O4—C8—C9	121.8 (2)
O4—Pb1—O1	121.06 (6)	O5—C8—C9	116.3 (2)
O5—Pb1—O1	132.63 (6)	C10—C9—C8	120.0 (2)
O5—Pb1—O4	50.94 (6)	C14—C9—C8	119.6 (2)
O5—Pb1—N1	77.24 (7)	C14—C9—C10	120.4 (3)
N1—Pb1—O1	79.96 (7)	C11—C10—C9	119.7 (3)
N1—Pb1—O4	125.57 (7)	C11—C10—H10	120.1
C1—O1—Pb1	83.85 (16)	C9—C10—H10	120.1
C1—O2—Pb1	103.41 (16)	O6—C11—C10	118.7 (3)
C4—O3—H31	105 (3)	O6—C11—C12	120.8 (3)
C8—O4—Pb1	84.76 (16)	C10—C11—C12	120.5 (3)
C8—O5—Pb1	102.42 (17)	C11—C12—H12	120.5
C11—O6—H61	116 (4)	C13—C12—C11	119.1 (3)
H82—O8—H81	92 (5)	C13—C12—H12	120.5
C15—N1—Pb1	124.95 (18)	C12—C13—H13	119.5
C19—N1—Pb1	116.64 (18)	C14—C13—C12	120.9 (3)
C19—N1—C15	118.1 (2)	C14—C13—H13	119.5
C20—N2—H21	120 (3)	C9—C14—H14	120.3
C20—N2—H22	120 (3)	C13—C14—C9	119.3 (3)
H22—N2—H21	119 (4)	C13—C14—H14	120.3
O1—C1—O2	121.7 (2)	N1—C15—C16	122.6 (3)
O1—C1—C2	121.4 (2)	N1—C15—H15	118.7
O2—C1—C2	116.9 (2)	C16—C15—H15	118.7
C3—C2—C1	119.8 (2)	C15—C16—C17	119.3 (3)
C3—C2—C7	120.2 (3)	C15—C16—H16	120.3
C7—C2—C1	120.0 (2)	C17—C16—H16	120.3
C2—C3—H3	120.0	C16—C17—C18	117.9 (2)
C4—C3—C2	120.0 (3)	C16—C17—C20	124.5 (3)
C4—C3—H3	120.0	C18—C17—C20	117.6 (3)
O3—C4—C3	121.7 (3)	C17—C18—H18	120.5
O3—C4—C5	118.3 (2)	C19—C18—C17	119.1 (3)
C3—C4—C5	119.9 (3)	C19—C18—H18	120.5
C4—C5—H5	119.9	N1—C19—C18	123.0 (3)
C6—C5—C4	120.1 (3)	N1—C19—H19	118.5
C6—C5—H5	119.9	C18—C19—H19	118.5
C5—C6—C7	120.2 (3)	O7—C20—N2	123.1 (3)
C5—C6—H6	119.9	O7—C20—C17	118.7 (3)
C7—C6—H6	119.9	N2—C20—C17	118.2 (3)
C2—C7—C6	119.6 (3)		
			
O2—Pb1—O1—C1	0.82 (14)	O1—C1—C2—C7	1.7 (4)
O4—Pb1—O1—C1	−35.34 (17)	O2—C1—C2—C3	2.1 (4)
O5—Pb1—O1—C1	28.21 (18)	O2—C1—C2—C7	−177.3 (2)
N1—Pb1—O1—C1	90.32 (16)	C4—C3—C2—C7	−0.1 (4)
O1—Pb1—O2—C1	−0.81 (14)	C4—C3—C2—C1	−179.5 (2)
O4—Pb1—O2—C1	148.04 (17)	C2—C3—C4—O3	178.6 (2)
O5—Pb1—O2—C1	−160.96 (16)	C2—C3—C4—C5	1.6 (4)
N1—Pb1—O2—C1	−83.29 (16)	C6—C5—C4—O3	−179.6 (2)
O1—Pb1—O4—C8	122.11 (16)	C6—C5—C4—C3	−2.4 (4)
O2—Pb1—O4—C8	94.12 (16)	C4—C5—C6—C7	1.8 (4)
O5—Pb1—O4—C8	0.14 (15)	C6—C7—C2—C1	178.8 (2)
N1—Pb1—O4—C8	21.70 (19)	C6—C7—C2—C3	−0.6 (4)
O1—Pb1—O5—C8	−99.11 (17)	C2—C7—C6—C5	−0.3 (4)
O2—Pb1—O5—C8	−78.09 (16)	O4—C8—C9—C10	−4.2 (4)
O4—Pb1—O5—C8	−0.13 (15)	O4—C8—C9—C14	176.1 (3)
N1—Pb1—O5—C8	−162.28 (17)	O5—C8—C9—C10	175.1 (2)
O1—Pb1—N1—C15	−132.2 (2)	O5—C8—C9—C14	−4.5 (4)
O1—Pb1—N1—C19	54.3 (2)	C8—C9—C14—C13	178.5 (3)
O2—Pb1—N1—C15	−80.7 (2)	C10—C9—C14—C13	−1.2 (5)
O2—Pb1—N1—C19	105.8 (2)	C11—C10—C9—C14	1.3 (4)
O4—Pb1—N1—C15	−11.0 (3)	C11—C10—C9—C8	−178.4 (2)
O4—Pb1—N1—C19	175.51 (18)	O6—C11—C10—C9	−179.8 (2)
O5—Pb1—N1—C15	6.0 (2)	C12—C11—C10—C9	−0.5 (4)
O5—Pb1—N1—C19	−167.5 (2)	O6—C11—C12—C13	178.9 (3)
Pb1—O1—C1—O2	−1.3 (2)	C10—C11—C12—C13	−0.4 (5)
Pb1—O1—C1—C2	179.7 (2)	C11—C12—C13—C14	0.5 (5)
Pb1—O2—C1—O1	1.6 (3)	C12—C13—C14—C9	0.3 (5)
Pb1—O2—C1—C2	−179.40 (18)	C17—C16—C15—N1	−1.4 (4)
Pb1—O4—C8—O5	−0.2 (2)	C18—C17—C16—C15	0.5 (4)
Pb1—O4—C8—C9	179.1 (2)	C20—C17—C16—C15	−179.4 (3)
Pb1—O5—C8—O4	0.3 (3)	C16—C17—C18—C19	0.3 (4)
Pb1—O5—C8—C9	−179.06 (19)	C20—C17—C18—C19	−179.8 (3)
Pb1—N1—C15—C16	−172.0 (2)	C16—C17—C20—O7	−178.6 (3)
C19—N1—C15—C16	1.5 (4)	C16—C17—C20—N2	1.9 (4)
Pb1—N1—C19—C18	173.3 (2)	C18—C17—C20—O7	1.5 (4)
C15—N1—C19—C18	−0.7 (4)	C18—C17—C20—N2	−177.9 (3)
O1—C1—C2—C3	−178.9 (2)	N1—C19—C18—C17	−0.2 (4)

Symmetry codes: (i) *x*−1, *y*, *z*; (ii) −*x*, −*y*+2, −*z*.

### Hydrogen-bond geometry (Å, º)

**Table:**

*D*—H···*A*	*D*—H	H···*A*	*D*···*A*	*D*—H···*A*
N2—H21···O4^iii^	0.84 (4)	2.21 (4)	3.046 (3)	175 (4)
N2—H22···O8^iv^	0.85 (4)	2.06 (4)	2.880 (5)	162 (4)
O3—H31···O7^v^	0.86 (5)	1.81 (5)	2.646 (3)	166 (4)
O6—H61···O1^vi^	0.73 (6)	2.05 (6)	2.755 (4)	161 (6)
O8—H81···O6^vii^	0.89 (4)	2.02 (4)	2.846 (3)	154 (4)
O8—H82···O2^viii^	0.87 (5)	2.31 (5)	3.018 (3)	139 (5)
O8—H82···O3^ix^	0.87 (5)	2.44 (6)	3.054 (3)	128 (4)
C12—H12···O1^vi^	0.93	2.57	3.269 (4)	132
C15—H15···O5	0.93	2.46	3.060 (4)	122
C16—H16···O8^iv^	0.93	2.51	3.413 (4)	165

Symmetry codes: (iii) *x*+1, *y*, *z*−1; (iv) *x*+1, *y*+1, *z*−1; (v) *x*, *y*, *z*+1; (vi) *x*, *y*+1, *z*; (vii) *x*, *y*−1, *z*; (viii) −*x*, −*y*+1, −*z*+1; (ix) −*x*+1, −*y*+1, −*z*+1.
